# Connectivity Recovery Based on Boundary Nodes and Spatial Triangle Fermat Points for Three-Dimensional Wireless Sensor Networks

**DOI:** 10.3390/s24247876

**Published:** 2024-12-10

**Authors:** Hongsheng Chen, Ke Shi

**Affiliations:** 1 College of Computer Science and Technology, Hubei University of Science and Technology, Xianning 437100, China; chenhs1981@163.com; 2 School of Computer Science and Technology, Huazhong University of Science and Technology, Wuhan 430074, China

**Keywords:** three-dimensional wireless sensor networks, connectivity recovery, spatial triangle Fermat points, heuristics algorithms, boundary nodes

## Abstract

In recent years, wireless sensor networks have been widely used, especially in three-dimensional environments such as underwater and mountain environments. However, in harsh environments, wireless sensor networks may be damaged and split into many isolated islands. Therefore, restoring network connectivity to transmit data effectively in a timely manner is particularly important. However, the problem of finding the minimum relay nodes is NP-hard, so heuristics methods are preferred. This paper presents a novel connectivity recovery strategy based on boundary nodes and spatial triangle Fermat points for three-dimensional wireless sensor networks. The isolated islands are represented as the boundary nodes, and the connectivity recovery problem is modeled as a graph connectivity problem. Three heuristics algorithms—the variant Kruskal algorithm, the variant Prim algorithm, and the spatial triangle Fermat point algorithm—are proposed to solve this problem. The variant Kruskal algorithm and the variant Prim algorithm connect the isolated islands by constructing the minimum spanning tree to link all the boundary nodes and placing relay nodes along the edges of this tree. We derive an accurate formula to determine the coordinates of spatial triangle Fermat points. Based on this formula, the spatial triangle Fermat point algorithm constructs a Steiner tree to restore network connectivity. Extensive simulation experiments demonstrate that our proposed algorithms perform better than the existing algorithm.

## 1. Introduction

Wireless sensor networks (WSNs) are distributed systems consisting of spatially distributed autonomous sensors equipped with wireless communication interfaces and sensing devices to cooperatively monitor physical or environmental conditions at different locations. This technology has become increasingly important in both military and civilian applications due to its ability to provide detailed, real-time information about the environment. As applications become more widespread, WSNs extend from traditional two-dimensional (2D) settings to more complex three-dimensional (3D) spaces.

This shift is particularly evident in the growth of underwater wireless sensor networks (UWSNs), which have been widely applied in scenarios such as marine scientific research, marine resource development, maritime search and rescue, ocean environmental monitoring, marine biological tracking, and pollution control. However, within UWSNs, communication between sensor nodes is facilitated through distinctive acoustic signals, whose transmission is affected by serious multipath effects, leading to severe propagation delays and high error rates. Underwater deployment of these sensor nodes also makes them susceptible to malfunction. Moreover, their limited energy supply results in the demise of some nodes as their battery power is exhausted, which causes energy-hole issues. To address these issues, Anwit R. et al. [1,2] proposed a heap-based energy replenishment method and a mobile sink trajectory optimizing method, respectively. On the other hand, due to the harsh underwater environment, some harmful factors may emerge, such as jellyfish, sharks, aggressive fish, boulders, unpredictable weather, and similar marine items, which will lead to node failure and network segmentation. Therefore, restoring network connectivity in time and transmitting data effectively in real-time when the network is disconnected is particularly important.

In addition to the UWSNs mentioned above, other networks, such as AANETs, collectively form a collaborative 3D network in land, sea, and air domains. This paper addresses the challenges of deploying sensor networks in battlefield 3D environments, such as underwater or mountainous terrains, prone to large-scale damage. Furthermore, the study also considers the scenario where drone swarms are susceptible to large-scale attacks, and the entire 3D network is severely damaged during disasters such as earthquakes or tsunamis. The research in this paper aims to investigate efficient methods for rapidly restoring network communication in such situations.

When the network is damaged on a large scale, the most critical problem is deploying or moving the least number of nodes to crucial locations to restore network connectivity. This problem has been proved to be an NP problem. Therefore, almost all the literature uses heuristic algorithms to find the deployment location when solving this problem, among which the Steiner Minimum Tree (SMT) algorithm is the most commonly used in 2D environments. This algorithm often degenerates into the Minimum algorithm Spanning tree based on a Single-tiered Relay Node Placement (MS_1TRNP) [3,4,5,6,7]. Moreover, connectivity restoration is particularly difficult in 3D WSNs, where the added dimension brings the added complexity of spatial relationships and complicates geometric computation and topological analysis.

However, more research is needed on connectivity recovery in a 3D environment. Most researchers still use traditional active clustering recovery algorithms to restore network connectivity. They use active recovery strategies that detect faults or vulnerabilities and then depend on fault recovery to fix the network [8,9,10,11,12,13,14,15,16,17]. This method is very effective for recovering network connectivity after a single node or a few nodes are damaged. However, the efficiency will be greatly reduced if a large-scale damaged network is divided into many islands.

As far as we know, Liu et al. [18] proposed an effective connectivity recovery strategy when a UWSN is damaged on a large scale and achieved good results. This strategy builds an adjustment tree by selecting Fermat points. However, when choosing the deployment node, this method determines the deployment position of the new node by adjusting the *Z* coordinate under the condition of determining the *X* coordinate and *Y* coordinate, and the value obtained when calculating the Fermat point is also an approximate value rather than an accurate value, so the result obtained is not optimal. At the same time, when defining the isolated island, one of the locations is also taken as the representative location, but the island isolation may be more significant in practice. This will also have an impact on the algorithm’s overall performance.

Kumar R. et al. [19] introduced a deployment strategy in a 3D setting that utilizes Delaunay tetrahedrons. Sensors are positioned at specific locations to guarantee network connectivity. The 3D space is divided into tetrahedron-shaped sectors where the nodes are placed. Experiments demonstrate that their approach is more efficient. However, they also use one location to represent one isolated island, and when deploying relay nodes, the *Z* coordinate of the location is also fixed. Thus, this result of the approach could be more optimal.

To restore network connectivity while the networks are damaged on a large scale in 3D WSNs, we propose a new strategy based on boundary nodes and spatial triangle Fermat points and verify it through three algorithms, namely, the variant Kruskal algorithm, the variant Prim algorithm, and the spatial triangle Fermat point algorithm. Our significant contributions are summarized as follows:

(1) The isolated islands are represented as the boundary nodes, and the connectivity recovery problem for 3D WSNs is modeled as a graph connectivity problem.
(2) We use 3D coordinates directly without additional constraints and derive the accurate calculation formula for the 3D coordinates of spatial triangle Fermat points.
(3) We propose three algorithms based on our constructed models and derived formulas: the variant Kruskal algorithm, the variant Prim algorithm, and the spatial triangle Fermat point algorithm. Experiments show that these three algorithms have achieved good results in the number of deployed nodes, average node degree, and other performance indicators.

The rest of this paper is organized as follows. Section 2 summarizes related works in connectivity recovery in 3D WSNs. In Section 3, we define the connectivity recovery problem and its model. The proposed algorithms and corresponding analysis are given in Section 4. Section 5 evaluates the performance of the proposed algorithms through simulation. Finally, Section 6 gives conclusions and directions for future research.

## 2. Related Work

There is much research on connectivity recovery, but more research on connectivity recovery in 3D WSNs is needed. Some traditional recovery algorithms in 2D environments are ineffective in 3D environments, so researchers continue to propose new recovery strategies. Of course, some researchers have improved recovery algorithms in 2D environments and achieved good results.

Based on the recent literature on connectivity recovery in 3D environments, underwater, aerial, and post-disaster scenarios are the primary application scenarios. In underwater scenarios, active recovery methods are predominantly employed, with connectivity being restored through the relocation and re-clustering of managed nodes. In aerial scenarios, passive strategies are prevalent, given that the aerial WSN is largely composed of Unmanned aerial vehicles (UAVs) and similar flying apparatus; many rely on mobile clustering for data transmission and the restoration of connectivity. In post-disaster scenarios, passive strategies are also commonly adopted, primarily using UAVs, other aerial devices or handheld equipment, and the limited remaining infrastructure to facilitate communication recovery. Some recovery algorithms in 3D environments use sleep scheduling and power adjustment to achieve connectivity recovery. Table 1 summarizes the methods mentioned in the current literature for comparison.

### 2.1. Connectivity Recovery in 2D WSNs

The connectivity recovery of wireless ad hoc/sensor networks in a 2D environment is still a hot topic. The literature [23,24,25,26,27,28,29,30,31,32] is the latest research in this area. Considering the aspect of timing, most studies continue to employ active, passive, or mixed active-passive approaches for network recovery, which rely on clustering and managed mobility to establish and maintain network connectivity.

(1) Pre-processed active recovery

This recovery method refers to all nodes in the network making some preparations before a node failure occurs. The aim is to restore connectivity immediately in case of node failure or network disconnection. The literature [28,31,32] adopts this method for fault tolerance recovery.

S. Darwish et al. [28] presented an adaptive cellular automata-based scheme for fault tolerance diagnosis and the preservation of network connectivity within WSNs. The literature [31] proposed an adaptive and fault-tolerant routing algorithm that uses particle swarm optimization to construct routing, including inter-cluster and intra-cluster structures, considering distance, energy, and traffic. Liu et al. [32] introduced a multipath reliable transmission algorithm known as BIM2RT, designed for the fault tolerance mechanism in WSNs, leveraging the best–worst ant system (BWAs) immune mechanism.

(2) Passive recovery

Passive recovery pertains to the processes and measures taken in response to node failures. In passive recovery, certain approaches incorporate pre-processing steps, including the preemptive evaluation of area geometry, training, and learning critical parameters, while others forgo the retention of any information. A method ensuring persistent connectivity (DBCE) in a heavily compromised sensor network environment was put forth in the literature [23], primarily designed to address subsequent damages. Liu et al. [25] introduced a machine learning-based connection recovery strategy (CRrbf) aimed at the industrial Internet of Things. To our knowledge, this represents one of the rare instances where machine learning is applied to the recovery of connections. Furthermore, the literature [27] proposed a dual connectivity recovery algorithm for WSN partitions, utilizing the principles of Steiner trees and convex polygons.

(3) Hybrid active/passive recovery

This type of recovery involves preemptive actions, including preserving data on neighboring nodes and following procedures in the event of node failure. Both references [24] and [26] utilize this hybrid recovery approach. These strategies are characterized as *K*-connected recovery algorithms, known for significantly enhancing network resilience. Reviews in references [29] and [30] summarize key technologies involving WSNs’ connectivity, coverage, and others.

All the above connectivity recovery methods deal with connectivity restoration in 2D WSNs. Although these methods are efficient in 2D WSNs, they are not necessarily applicable in 3D WSNs.

### 2.2. Connectivity Recovery in 3D WSNs

Connectivity recovery is very important in 3D WSNs because it can ensure the data transmits effectively in time. Many methods proposed for connectivity recovery adopt active recovery strategies, from fault detection or vulnerability detection to fault recovery.

The literature [8,9,10,11,12,13,14,15,16,17] explores active clustering methods for connectivity recovery of UWSNs. The literature [8,9] proposed a coverage vulnerability repairing algorithm based on clustering and sleep scheduling in underwater wireless sensor networks. Chaaf A. et al. [12] also proposed a clustered multi-autonomous underwater vehicle (multi-AUV) vulnerability prediction and repairing method (RevoHPR) in UWSNs, which introduces multiple autonomous underwater vehicles to establish a stable clustering structure.

Reference [10] introduced an autonomous recovery approach named CSO, which is based on cat group optimization and specifically designed for heterogeneous UWSNs. Reference [13] proposed a hidden Poisson Markov model for detecting node failures and the subsequent recovery algorithms. Reference [14] proposed a fault prediction, detection, and recovery algorithm that operates on a tree network topology, employing the Markov chain Monte Carlo (MCMC) process. References [16,17] proposed recovery algorithms centered around cluster-based methodologies, utilizing cluster head and candidate head roles in the recovery process. Lastly, reference [11] presented an anomaly detection technique tailored for UWSNs. This algorithm mainly represents the routing protocol by using the detection algorithm and modifying the routing path in UWSNs, reducing the number of damaged sensors.

Reference [15] has put forward a node-sinking algorithm to enhance the 3D coverage and connectivity of UWSNs. Reference [20] has introduced a priority-based strategy for coverage hole restoration and ensuring *m*-connection, utilizing a whale optimization algorithm. Performance assessments indicate that this proposed approach surpasses existing methods across diverse network configurations, offering superior coverage and connectivity, minimizing energy consumption, and attaining a rapid convergence rate. However, it overlooks aspects such as barrier coverage holes and the complexities of heterogeneous underwater network environments during the connectivity coverage process.

Reference [21] introduces a distributed hole detection and multi-objective optimization-based Emperor Penguin Repair algorithm (DHD-MEPO), designed to tackle the challenge of coverage holes in 3D hybrid WSNs. In their algorithm, they consider the number of nodes, the sensing range, the sum-of-weights of nodes, the mobile node coverage redundancy, and the residual energy homogeneity of nodes. Nevertheless, in practical settings, the sensing range of sensor nodes is influenced by environmental fluctuations and the presence of multiple barriers, which causes the actual value to change over time.

Reference [22] has developed a coverage and connectivity-aware deployment strategy for optimal positioning of AUVs within UWSNs. This strategy introduced a novel fitness function encompassing the quality of coverage, the cost of connectivity, and the duration of the network’s lifespan. This proposed scheme also employs an efficient encoding method for representing the population. Simulation results demonstrate that the proposed method effectively improves network coverage. However, this technique is unsuitable for heterogeneous UWSNs, where different AUVs may have different sensing and communication capabilities.

These connectivity recovery techniques address the restoration of connectivity in 3D WSNs. Most of the literature relies on active clustering and node mobility for restoration, which involves two primary stages: fault detection and the actual recovery of connectivity. Some studies also integrate sleep scheduling and power adjustment to maintain network connectivity. Network connectivity restoration through deploying new nodes is challenging, especially in underwater settings, with most methods utilizing underwater vehicle movement for data collection and network reconnection.

To our knowledge, only Liu et al. [18] and Kumar R et al. [19] have proposed strategies for connectivity recovery in 3D WSNs. Liu et al. [18] suggested an effective strategy for large-scale damage in UWSNs, but their algorithm has limitations, primarily because it considers only one node in an isolated network partition as the representative node during modeling. The *X* and *Y* coordinates are fixed when deploying relay nodes, which may not yield the most optimal solution. Kumar R et al. [19] introduced a Delaunay tetrahedron-based deployment strategy in a 3D WSN, focusing on networks divided into several sub-networks due to various factors. The 3D space between these divisions is triangulated using Delaunay tetrahedrons, with the circumcenter of each tetrahedron being a potential node placement position. However, their method also uses a single location to represent an isolated network partition, and the *Z* coordinate is fixed when deploying relay nodes, which could potentially limit the optimality of their approach.

From the analysis above, existing methods that deploy relay nodes to restore network connectivity determine the deployment location of relay nodes based on specific spatial partitioning strategies. To reduce computational complexity, these methods have been simplified, often failing to find the optimal location and thus increasing the number of relay nodes. Our proposed methods adopt the boundary node detection algorithm to find the appropriate node sets to represent the disjoint sub-networks and construct the minimum spanning tree or Steiner tree to connect them. When constructing the Steiner tree, the formula for determining the 3D coordinates of the spatial triangle Fermat point is derived. The damaged network can be restored with a smaller number of relay nodes.

## 3. Model and Problem Definition

### 3.1. System Model

The WSNs studied in this paper are deployed underwater, in forests, mountains, mines, and other harsh 3D environments to monitor the designated area. The sensor nodes are static, and their sensing radius and communication radius are fixed. Sensor nodes can communicate with each other if they are within their communication radius, and finally transmit the collected data to the base station for corresponding processing. Sensor nodes can obtain their own location information through the relevant positioning algorithm. The whole network adopts a hierarchical topological structure of clustering.

**Figure 1:**
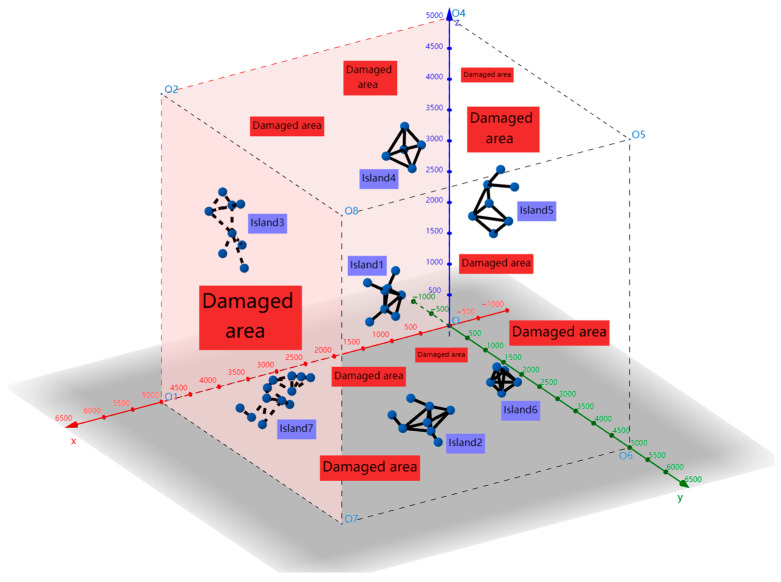
Severely damaged 3D wireless sensor network.

Generally, sensor nodes may fail due to energy depletion, but only a few will fail in most cases. No processing can be performed if it does not affect the network connectivity. Otherwise, it is only necessary to find the corresponding failure node through the detection algorithm and rearrange a small number of nodes. However, in the harsh 3D environments that this paper focuses on, in addition to energy depletion failure, sensor nodes will also have large-scale failure due to the impact of the external environment (such as shark attacks, unpredictable weather, etc.). The entire network will be damaged in this case, as shown in Figure 1.

In Figure 1, the blue dots represent sensor nodes, the black lines indicate connectivity between them, and connected nodes form an isolated island. In Figure 1, there are seven isolated islands, while the other areas represent damaged areas.

When the sensor network is divided into multiple isolated islands, the sensor nodes can obtain information about other nodes on the same island through mutual communication and obtain each island’s set of boundary nodes through the boundary acquisition algorithm presented in the literature [33]. The network connectivity problem is abstracted as a graph connectivity problem, i.e., how to find a method to deploy a certain number of relay nodes to connect *n* islands with *n* boundary node sets.

To solve this problem, the global island information of the network is required. Therefore, the island’s information needs to be transmitted to the base station with the help of specific patrol equipment, and the base station can restore the network connection. Regular inspection is necessary and feasible in many practical applications, such as inspection of underwater vehicles, regular personnel inspection in ecological environment monitoring, and regular surveillance of UAVs on the battlefield. Thus, underwater vehicles, personnel handheld devices, and UAVs can serve as patrol equipment to collect global information about the network.

### 3.2. Problem Definition

By constructing the above model, the connectivity restoration problem of 3D WSNs can be abstracted as the connectivity problem of the graph.

**Definition 1.**  *Island. When severe damage occurs, multiple nodes fail at the same time, which causes the WSN to break into disjoint sub-networks. Island refers to a disjoint sub-network.*

If the nodes are on the same island, they can communicate; if the nodes are on different islands, they cannot. Therefore, these remaining sub-networks, after severe damage, are independent of each other. They cannot perform the planned functions, such as data acquisition and transmission, regardless of the number of base stations in the original WSN.

**Definition 2.**  *Boundary node-set. It refers to the set of boundary nodes in the network corresponding to the island i, which is represented by* $U^{i}$*. The boundary node set of all islands in the network is represented by* U*, that is,* $U=U^{1}\cup U^{2}\cup \ldots \cup U^{n},\;i=1,\;2,\;\ldots ,\;n$*, where n represents the number of islands.*

Therefore, the goal of this problem is to connect the islands into a connected graph by constructing the minimum spanning tree and the spatial triangle Fermat point Steiner tree, which is represented by $G=\left(V,\;E\right)$. In the model proposed in this paper, *V* includes two types of nodes: boundary nodes and deployed relay nodes. *E* refers to the set of edges connecting the above two types of nodes, assuming that the length of each edge is represented by $\left|v_{i}v_{j}\right|$. Then the problem can be defined as follows:

Minimize $\sum_{E}\left|v_{i}v_{j}\right|$, subject to: G is connected

Where *E* represents the set of edges in a connected graph G, and $v_{i}$ and $v_{j}$ are two nodes in set *V*. That is, $v_{i}$ and $v_{j}$ are either boundary nodes in islands or relay nodes.

## 4. Connectivity Recovery Strategy Combining Boundary Nodes and Spatial Triangle Fermat Points

The strategy proposed in this paper first detects islands, determines the boundary nodes and their positions of each island, and then transmits the information about each island and boundary node to the base station through patrol equipment. Finally, according to the collected location information of each island and its boundary nodes, the variant Kruskal algorithm, variant Prim algorithm, and spatial triangle Fermat algorithm are used to connect the islands and restore the connectivity of the entire network.

### 4.1. Basic Theory of Algorithm

The basic theory and related terms of the Fermat point used in this paper are as follows:

**Definition 3.**  *Steiner point: To construct a spanning tree connecting n points in the plane, intermediate points need to be included to reduce the edge length. These intermediate points that reduce the size of the spanning tree are called Steiner points. The resulting tree is called a Steiner tree. A minimal Steiner tree minimizes total edge length.*

**Definition 4.**  *Fermat point: It refers to the point located inside a triangle where the sum of its distances to the triangle’s three vertices is minimized.*

**Definition 5.**  *Spatial triangle: It refers to a triangle in 3D space; that is, the coordinates of three points of a triangle are 3D coordinates.*

**Definition 6.**  *Fermat point of the spatial triangle: If the three internal angles of the spatial triangle are less than 120°, a point must be found inside the triangle. The included angle between this point and the three vertices of the triangle is 120°, and the sum of the distances between this point and the three vertices is the smallest. This point is the Fermat point of the spatial triangle. If an internal angle of a triangle is greater than 120°, the obtuse angle vertex is a Fermat point.*

**Figure 2:**
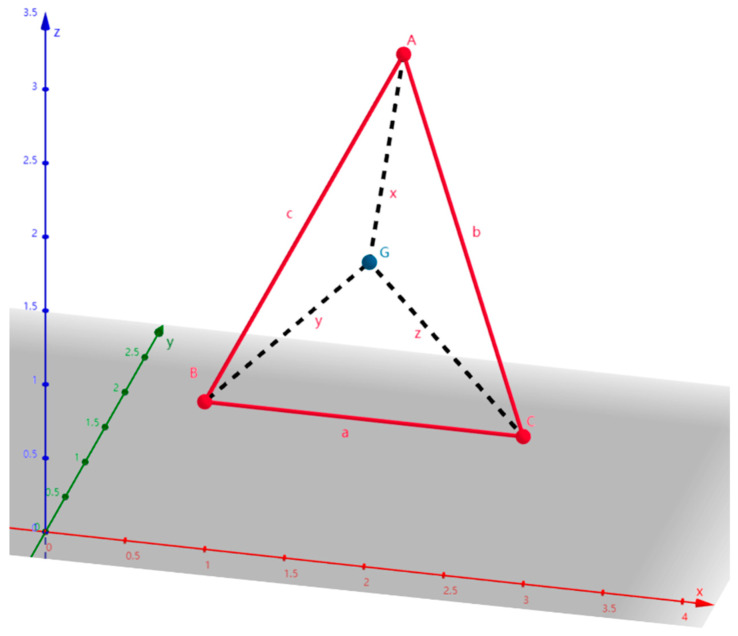
An example of a spatial triangle.

To find the Fermat point, the specific definition is given in the plane triangle, and the corresponding solution formula is developed. However, as far as we know, how to find the Fermat point of a 3D space triangle has yet to be addressed in the literature. This paper develops a method to determine the 3D coordinates of the spatial triangle Fermat point and gives the particular solution formula. The particular derivation process is as follows:

First, a spatial triangle is given, as shown in Figure 2. Let *A*, *B*, and *C* be the three vertices of the spatial triangle, and *G* (*p*, *q*, *r*) be the Fermat point of the spatial triangle. The coordinates of *A*, *B*, and *C* are *A* (*x*_1_, *y*_1_, *z*_1_), *B* (*x*_2_, *y*_2_, *z*_2_), and C (*x*_3_, *y*_3_, *z*_3_), and the lengths of line segments are *AG* = *x*, *BG* = *y*, *CG* = *z, AB* = *c*, *AC* = *b*, and *BC* = *a*.

According to the property of Fermat point: ∠*AGB* = ∠*BGC* = ∠*AGC* = 120°, the following formula is obtained from the cosine theorem:

*a*^2^ = *y*^2^ + *z*^2^ − 2*yz*
*cos*120° ⇒ *y*^2^ + *z*^2^ + *yz* = *a*^2^ (1)

*ditto*: *x*^2^ + *z*^2^ + *xz* = *b*^2^ (2)

*x*^2^ + *y*^2^ + *xy* = *c*^2^ (3)

Then,

(4) $$S\Delta AGB=\frac{1}{2}*sin120{}^{\circ}*xy=\frac{\sqrt{3}}{4}*xy$$

(5) $$S\Delta AGC=\frac{\sqrt{3}}{4}*xz$$

(6) $$S\Delta BGC=\frac{\sqrt{3}}{4}*yz$$

From Equations (4)–(6), we can obtain:

(7) $$S\Delta ABC=\frac{\sqrt{3}}{4}*\left(xy+yz+xz\right)=S\;\;\Rightarrow xy+yz+xz=\frac{4}{3}\sqrt{3}*S$$

where *S* is the area of Δ*ABC*.

From Equations (1)–(3), we can obtain:

(8) $$2x^{2}+2y^{2}+2z^{2}+xy+yz+xz=a^{2}+b^{2}+c^{2}$$

Dividing both sides of Equation (8) by 2 yields the following Equation (9):

(9) $$x^{2}+y^{2}+z^{2}+\frac{1}{2}*xy+\frac{1}{2}*yz+\frac{1}{2}*xz=\frac{1}{2}*\left(a^{2}+b^{2}+c^{2}\right)$$

Adding $\frac{3}{2}*\left(xy+xz+yz\right)$ to both sides of Equation (9) and dividing it by 2 yields the following Equation (10):

(10) $$x^{2}+y^{2}+z^{2}+2xy+2xz+2yz=\frac{3}{2}*\left(xy+xz+yz\right)+\frac{1}{2}*\left(a^{2}+b^{2}+c^{2}\right)$$

Expanding the left-hand side of Equation (10) yields the following Equation (11):

(11) $${\left(x+y+z\right)}^{2}=\frac{3}{2}*\left(xy+xz+yz\right)+\frac{1}{2}*\left(a^{2}+b^{2}+c^{2}\right)$$

Substituting Equation (7) into Equation (11) yields Equation (12).

(12) $$\left(x+y+z{)}^{2}=2\sqrt{3}*S+\frac{1}{2}*(a^{2}+b^{2}+c^{2}\right)$$

Let

(13) $$\lambda^{2}=2\sqrt{3}*S+\frac{1}{2}*\left(a^{2}+b^{2}+c^{2}\right)$$

We can obtain

(14) x+y+z=λ

Then, combining Equation (14) with (1)–(3) to obtain:

(15) $$x=\frac{\left(b^{2}+c^{2}-2a^{2}+\lambda^{2}\right)}{3\lambda }$$

(16) $$y=\frac{\left(a^{2}+c^{2}-2b^{2}+\lambda^{2}\right)}{3\lambda }$$

(17) $$z=\frac{\left(a^{2}+b^{2}-2c^{2}+\lambda^{2}\right)}{3\lambda }$$

According to Helen’s formula:

(18) $$S=\frac{1}{4}*\sqrt{\left(a+b+c\right)*\left(a+b-c\right)*\left(a+c-b\right)*\left(b+c-a\right)}$$

Substituting Equation (18) into Equation (13) to obtain λ, then substituting λ to Equations (15)–(17), we can obtain the values of *x*, *y*, and *z*.

The following equations are obtained from the distance formula between two points:

(19) $$(x_{1}-p{)}^{2}+(y_{1}-q{)}^{2}+(z_{1}-r{)}^{2}=x^{2}$$

(20) $$(x_{2}-p{)}^{2}+(y_{2}-q{)}^{2}+(z_{2}-r{)}^{2}=y^{2}$$

(21) $$(x_{3}-p{)}^{2}+(y_{3}-q{)}^{2}+(z_{3}-r{)}^{2}=z^{2}$$

From Equations (19)–(21) we can obtain the following equations:

(22) $${x_{1}}^{2}-2x_{1}p+p^{2}+{y_{1}}^{2}-2y_{1}q+q^{2}+{z_{1}}^{2}-2z_{1}r+r^{2}=x^{2}$$

(23) $${x_{2}}^{2}-2x_{2}p+p^{2}+{y_{2}}^{2}-2y_{2}q+q^{2}+{z_{2}}^{2}-2z_{2}r+r^{2}=y^{2}$$

(24) $${x_{3}}^{2}-2x_{3}p+p^{2}+{y_{3}}^{2}-2y_{3}q+q^{2}+{z_{3}}^{2}-2z_{3}r+r^{2}=z^{2}$$

The *p*^2^, *q*^2^, and *r*^2^ are eliminated by (22)–(23), (22)–(24), and (23)–(24):

(25) $$\left({x_{1}}^{2}-{x_{2}}^{2}\right)+2\left(x_{2}-x_{1}\right)p+\left({y_{1}}^{2}-{y_{2}}^{2}\right)+2\left(y_{2}-y_{1}\right)q+\left({z_{1}}^{2}-{z_{2}}^{2}\right)+2\left(z_{2}-z_{1}\right)\;r=x^{2}-y^{2}$$

(26) $$\left({x_{1}}^{2}-{x_{3}}^{2}\right)+2\left(x_{3}-x_{1}\right)p+\left({y_{1}}^{2}-{y_{3}}^{2}\right)+2\left(y_{3}-y_{1}\right)q+\left({z_{1}}^{2}-{z_{3}}^{2}\right)+2\left(z_{3}-z_{1}\right)\;r=x^{2}-z^{2}$$

(27) $$({x_{2}}^{2}-{x_{3}}^{2})+2(x_{3}-x_{2})p+({y_{2}}^{2}-{y_{3}}^{2})+2(y_{3}-y_{2})q+({z_{2}}^{2}-{z_{3}}^{2})+2(z_{3}-z_{2})\;r=y^{2}-z^{2}$$

Then, the Fermat point coordinates *G* (*p*, *q*, *r*) of the spatial triangle can be obtained by Equations (25)–(27).

### 4.2. Proposed Algorithms

#### 4.2.1. Variant Kruskal Algorithm

The variant Kruskal algorithm proposed in this paper mainly uses the classic Kruskal algorithm as a reference and improves it by combining it with the model proposed in this paper.

The pseudo-code of the variant Kruskal algorithm is shown in Algorithm 1. It contains the following steps:

Step 1: Detected the set of boundary nodes of each island through the boundary acquisition algorithm in the literature [33]. Put the boundary nodes of each detected island into a set $U^{\prime }$. Supposing there are *n* islands, all the island boundary sets form a large set U.

Step 2: Find the two nodes with the shortest distance in $U^{\prime }$, and these two nodes must be in different $U^{\prime }$; that is, on different islands.

Step 3: After connecting the two nodes, the two sets corresponding to the two nodes are merged into a new set $U^{\prime \prime }$, which is added to set U, and the original set corresponding to the two nodes is deleted in $U^{\prime }$.

Step 4: Continue to perform steps 2–3 until all sets are merged into only one set.

Step 5: Finally, relay nodes are deployed along each path found according to the transmission radius of relay nodes to connect the whole network.
**Algorithm 1** Variant Kruskal algorithm (VKA)**Input:** The number of the island *n*, the number of the boundary node of each island *m*, the 3D coordinates of each boundary node, the transmission radius *r* of the relay node, the area length, width, and height **Output:** The number of relay nodes, the average node degree, the average hops, and the coordinates of the deployment node1: **while** There are Islands **do** 2:    island1, island2 (*I1*, *I2*) ← NONE 3:    Minimal Island distance (*Mid*) ← ∞ 4:    **for** item1 = the first island to the penultimate island **do** 5:     **for** item2 = next of item1 to the last island **do** 6:      **if** distance from item1 to item2 < *Mid* **then** 7:       *Mid* = distance from item1 to item2 8:       *I1* = item1 9:       *I2* = item2 10:    **end if** 11:   **end for** 12:  **end for** 13:  merge *I1* and *I2* 14: **end while**

**Theorem 1.**  *The time complexity of the variant Kruskal algorithm is O(n^3^ × m^2^), in which n represents the number of islands, and m represents the number of boundary nodes in each island.*

**Proof.**  In line 1, the main task is to determine the existence of isolated islands, which takes *n −* 1 iterations to eliminate all isolated islands. Therefore, the time complexity of this algorithm line is O(*n*). The fourth line involves traversing all isolated islands, resulting in a time complexity of O(*n*^2^). In line 5, it still involves traversing all isolated islands in the inner loop, so the time complexity is O(*n*^3^) for this part of the algorithm. The sixth line calculates the distance between isolated islands, resulting in a time complexity of O(*n*^3^ × *m*^2^). In line 13, the merging of isolated islands takes place, and the time complexity is O(*n*^3^ × *m*^2^ + *n* × *m*). Based on the above analysis, the time complexity of the VKA is O(*n*^3^ × *m*^2^). □

#### 4.2.2. Variant Prim Algorithm

The variant Prim algorithm proposed in this paper mainly uses the classic Prim algorithm as a reference and improves it by combining it with the model proposed in this paper.

The pseudo-code of the variant Prim algorithm is shown in Algorithm 2. It contains the following steps:

Step 1: Detected the set of boundary nodes of each island through the boundary acquisition algorithm in the literature [33]. Put the boundary nodes of each detected island into a set $U^{\prime }$. Suppose there are *n* islands, and all the island boundary sets form a large set U. Choose one island boundary set $U^{\prime }$ from U.

Step 2: Find the two nodes with the shortest distance between the nodes in $U^{\prime }$ and the nodes in other sets.

Step 3: After connecting the two nodes, the two sets corresponding to the two nodes are merged into a new set $U^{\prime \prime }$, which is added to set U, and the original set corresponding to the two nodes is deleted in U.

Step 4: Replace $U^{\prime }$ with $U^{\prime \prime }$. Continue to perform steps 2–3 for *n* − 1 times before ending.

Step 5: Finally, relay nodes are deployed along each path found according to the transmission radius of relay nodes to connect the whole network.
**Algorithm 2** Variant Prim algorithm (VPA)**Input:** The number of the island *n*, the number of the boundary node of each island *m*, the 3D coordinates of each boundary node, the transmission radius *r* of the relay node, and the area length, width, and height **Output:** The number of relay nodes, the average node degree, the average hops, and the coordinates of the deployment node1: Select an island(*Si*) as the starting point 2: island ← NONE 3: **while** there are islands **do** 4:   Minimal island distance (Mid) ← ∞ 5:   **for** item = the first island to the last island **do** 6:     **if** item is *Si* then **continue** 7:    **end if** 8:     **if** distance from item to *Si* < *Mid* **then** 9:        *Mid* ← distance from item to *Si* 10:      island ← item 11:   **end if** 12:  **end for** 13:  merge *Si* and island 14:  *Si* ← merged island 15: **end while**

**Theorem 2.**  *The time complexity of the variant Prim algorithm is O(n^2^ × m^2^), in which n represents the number of islands, and m represents the number of boundary nodes in each island.*

**Proof.**  In line 1, the selection of the starting point is arbitrary and direct, thus the time complexity is O(1). In line 4, the main task is to determine the existence of isolated islands, which takes *n* − 1 iterations to eliminate all isolated islands. Therefore, the time complexity of this algorithm line is O(*n*). The third line involves traversing all isolated islands, resulting in a time complexity of O(*n*^2^). The fifth line calculates the distance between isolated islands, resulting in a time complexity of O(*n*^2^ × *m*^2^). In line 13, the merging of isolated islands takes place, and the time complexity is O(*n*^2^ × *m*^2^ + *n* × *m*). Based on the above analysis, the time complexity of the VPA is O(*n*^2^ × *m*^2^). □

#### 4.2.3. Spatial Triangle Fermat Algorithm

The basic idea of the spatial triangle Fermat algorithm is to construct a spatial triangle and calculate the corresponding Fermat point to connect the separated islands. The disjoint islands that are not connected by these selected spatial triangles are connected with the variant Kruskal method. Finally, relay nodes are deployed to the appropriate position according to the edges of the Steiner tree to restore network connectivity.

The pseudo-code of the spatial triangle Fermat algorithm is shown in Algorithm 3. It contains the following steps:

Step 1: Detected the set of boundary nodes of each island through the boundary acquisition algorithm in the literature [33]. Put the boundary nodes of each detected island into a set $U^{\prime }$. Suppose there are *n* islands, and all the island boundary sets form a large set U. Randomly select three nodes in the boundary set U to form a spatial triangle, which should satisfy the following conditions:

(1) The three vertices must be in different boundary sets, that is, on other islands.
(2) The spatial triangle does not contain other nodes except three vertices.

Step 2: Judge whether the internal angles of this triangle are all less than 120°. If not, the Fermat point is the corresponding vertex of the obtuse angle.

Step 3: Otherwise, the Fermat coordinates of this spatial triangle are calculated by Formula (25)–(27), and relay nodes are deployed at corresponding locations along the connection between this Fermat point and the other three vertices according to the transmission radius of the relay node. The set of three vertices corresponding to this spatial triangle is merged into a new set $U^{\prime \prime }$ and added to U, and the original set corresponding to the three vertices is deleted simultaneously.

Step 4: Continue to perform steps 1–3.

Step 5: If a set is finally formed, the whole network will be connected; if not, the remaining set will be connected with the variant Kruskal algorithm to finally realize the connection of the whole network.
**Algorithm 3** Spatial triangle Fermat algorithm (STFPA)**Input:** The number of the island *n*, the number of the boundary node of each island *m*, the 3D coordinates of each boundary node, the transmission radius *r* of the relay node, and the area length, width, and height **Output:** The number of relay nodes, the average node degree, the average hops, and the coordinates of the deployment node1: merged islands(*Mi*) ← Minimal subscript island(*Msi*) 2: islands to be merged(*Itbm*) ← all islands except *Msi* 3: **while** *Itbm* presence island **do** 4:   Find the two islands(*I1*, *I2*) closest to *Msi* 5:   Find an island(*I3*) from Mi closest to *I1* or *I2* 6:   The shortest perimeter triangle(*Spt*) ← ∞ 7:   **for** item1 = first border node at *I1* to last border node at *I1* **do** 8:    **for** item2 = first border node at *I2* to last border node at *I2* **do** 9:     **for** item3 = first border node at *I3* to last border node at *I3* **do** 10:    **if** the perimeter of a triangle composed of *I1*, *I2*, *I3* < *Spt* **then** 11:     record *I1*, *I2*, *I3* 12:    **end if** 13:     **end for** 14:    **end for** 15:   **end for** 16:   use Fermat point links *I1*, *I2*, *I3* 17:   add *I1* and *I2* to *Mi*, and remove them from *Itbm* 18: **end while**

**Figure 3:**
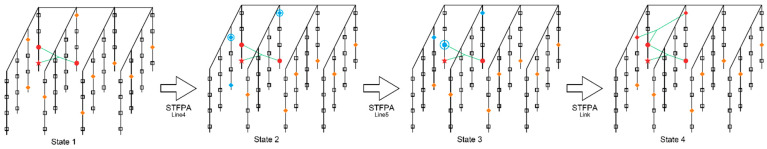
The process of constructing the second spatial triangle.

To better understand the STFPA, Figure 3 shows the general process of the second connection, which involves constructing the second spatial triangle. The white square in the diagram represents the damaged area; the diamond represents the island (that is, *Itbm*), and the pentagram represents *Msi*. If the diamond is orange, the corresponding island is isolated and needed to be re-connected. The green lines connect islands to form *Mi*, and the circle represents part of the network. If the circle is blue, it means the corresponding part has an opportunity to be re-connected. If the circle is red, it means the corresponding part has been re-connected. State1 represents the state of the environment after the first connection, that is, after constructing the first spatial triangle. State 2 indicates that three islands close to *Msi* are found. These three islands change from orange to blue, and finally, the two closest ones are selected as *I1* and *I2*, and the figure is circled in blue. State3 indicates finding *I3* from *Mi*, closest to *I1* and *I2*. The red circle turns blue and is highlighted. State4 represents the state of the environment after the second connection is completed, that is, the second spatial triangle is constructed.

**Theorem 3.**  *The time complexity of the spatial triangle Fermat algorithm is O(n × m^3^ + n^2^), in which n represents the number of islands, and m represents the number of boundary nodes in each island.*

**Proof.**  In line 3, the main task is to determine the existence of isolated islands; after *n*/2 floor divisions, there are no more isolated islands. Therefore, the time complexity of this algorithm line is O(*n*). In lines 4–5, the calculations are performed directly, resulting in a time complexity of O(1) for this part. Therefore, the overall time complexity is O(*n*). In lines 7–9, each line involves traversing the boundary nodes, resulting in a time complexity of O(*n* × *m*^3^) for this part of the algorithm. In line 13, the primary operations involve calculating the Fermat point of a triangle and performing coordinate calculations. The computational complexity for these calculations is O(1), while the time complexity for the connection is O(*n* × *m*^3^ + *n*). In line 14, removing the isolated island from the set results in a time complexity of O(*n* × *m*^3^ + *n* + *n*^2^) for this part of the algorithm. Based on the above analysis, the time complexity of the STFPA is O(*n* × *m*^3^ + *n*^2^). □

## 5. Performance Evaluation and Analysis

In this section, we will evaluate and analyze our proposed algorithms, namely, the variant Kruskal algorithm (VKA), the variant Prim algorithm (VPA), and the spatial triangle Fermat point algorithm (STFPA) by comparing their performance in terms of the number of relay nodes, the average node degree, and the average hops. To the best of our knowledge, ATCFS [18] is the latest algorithm proposed to address the connectivity restoration of 3D WSNs. We compared our algorithm with it. We implement all these algorithms in a simulated environment. The underlying WSNs are randomly generated from a random graph model.

In simulation, we mainly consider the following performance metrics and parameters:

(1) The number of islands: With more islands, more work is required for connectivity recovery, and more relay nodes are needed.
(2) Communication radius: The communication radius greatly impacts the performance of the algorithm proposed in this paper. The larger the communication radius is, the fewer nodes are needed to connect each island, and vice versa.
(3) The number of relay nodes: The relay node is deployed to achieve network connectivity. Obviously, under the condition of ensuring connectivity, the fewer relay nodes are used, the better the algorithm is.
(4) The average node degree: The average node degree refers to the average number of other nodes connected by each node. Obviously, the greater the average node degree is, the better the network’s robustness is.
(5) The average hop count: This performance metric is mainly used to evaluate whether the algorithm is good or bad by the number of forwarding nodes in the data transmission; the smaller the average hop count is, the more optimal the algorithm is.

### 5.1. Simulation Environment

**Figure 4:**
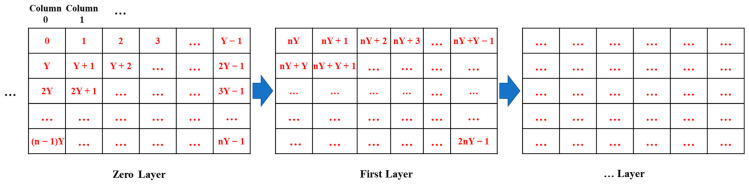
The process of partitioning the 3D space and emulating the generation of isolated islands.

To simulate a 3D WSN environment, we assume that the space size is 5000 m long, 5000 m wide, and 5000 m high. We divided the entire 3D space into small spaces representing islands or gaps. The partitioning process divides the entire 3D WSN space into a multiple hierarchical structure, with *Y* spaces in each row and *n* spaces in each column. The spaces are directly labeled with red numbers, as shown in Figure 4. We can observe the following mathematical patterns in labeling any space *a* and its adjacent spaces. From a top-down perspective in the same layer, we can see that the labels of the left/right spaces of space *a* are *a*’s label minus/plus 1, while the labels of the upper/lower spaces are *a*’s label minus/plus *Y*. From a multi-layer perspective, we can see that the labels of the upper/lower spaces are *a*’s label minus/plus *nY*. Therefore, we can directly calculate the positions of any other space relative to space *a*. The specific process is shown in Figure 4.

The nodes communicate via an omnidirectional, fixed-range, symmetric radio transmitter. This model has been widely used in 3D WSNs. Omnidirectional transmission means nodes transmit in all directions with equal power strength. Fixed-range transmission means all transmitters have a common, fixed power setting, translating into a fixed communication radius. Symmetric communication means if a node *v* can receive transmissions from a node *u*, then *v* also has enough transmission power to transit to node *u*. If a node needs to transmit to another node out of its radio range, the message must be relayed multi-hops by intermediate nodes. A node’s transmission range is modeled by a sphere. At the sphere’s center is the node and the radius is the transmission range. The initial node’s communication radius is 500 m and can be changed in the experiments. When the distance between nodes is greater than or equal to the communication radius, the nodes cannot communicate with each other, and vice versa, they can communicate. To simulate different environmental conditions, there are two types of space division.

**Figure 5:**
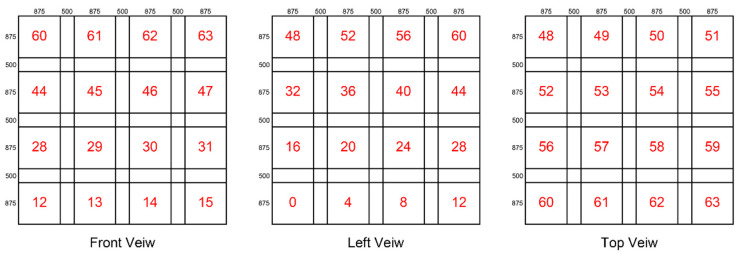
The first type of spatial division.

In the first type of space division, the size of an island space is 875 m × 875 m × 875 m, and each island space is 500 m apart. Figure 5 gives a division example displayed in 3D views, where the island spaces are numbered 0, 1, 2, …, 62, and 63, respectively.

**Figure 6:**
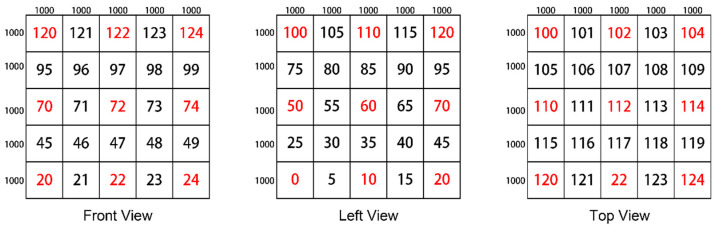
The second type of spatial division.

In the second type of space division, the size of an island space is 1000 m × 1000 m × 1000 m, and each island space is 1000 m apart. Figure 6 gives a division example displayed in 3D views, where the island spaces are numbered as 0, 2, 4, 10, 12, 14, 20, 22, 24, 50, 52, 54, 60, 62, 64, 70, 72, 74, 100, 102, 104, 110, 112, 114, 120, 122, and 124, respectively. These labels are marked with red colors. The rest of the spaces with black labels are the gaps separating these island spaces.

After the space division, we randomly select a subset of the island spaces and put sensors in these spaces. For a selected island space, a set of points is generated randomly to represent the sensors’ locations. Then, the boundary node of this island is determined. After the required number of boundary nodes reaches the experimental specifications, the degree of each boundary node is determined based on the distance between sensors. Therefore, the second division type simulates the scenario in which the sensor networks are damaged more severely, and the distribution of partitions is sparser.

### 5.2. Simulation Result

For all the simulations, we repeat the experiment 1000 times and report the average values of the metric.

#### 5.2.1. The Performance Comparison of the Number of Relay Nodes

**Figure 7:**
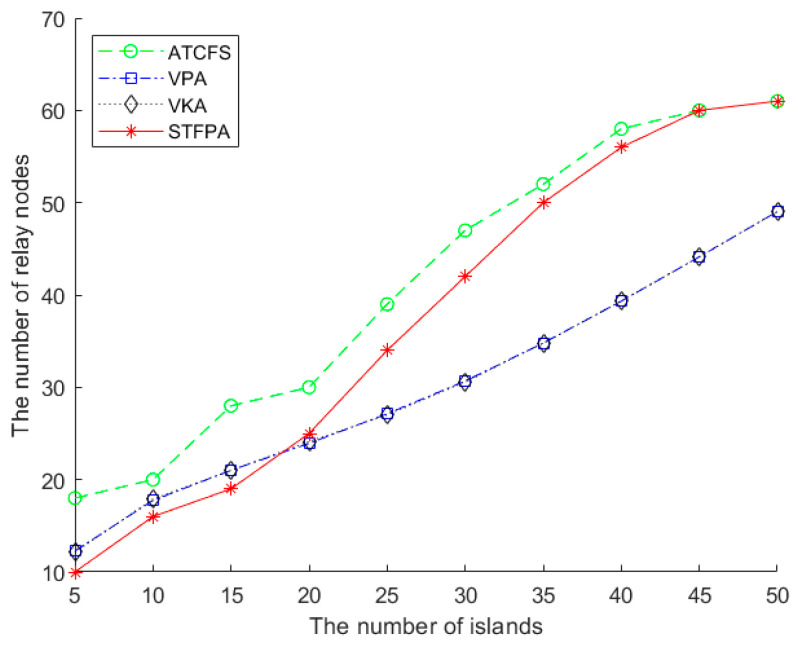
The number of relay nodes vs. the number of islands.

Figure 7 shows the impact of the number of islands on the number of relay nodes when there are 20 boundary sensors in one island, and the communication radius is 500 m. As shown in Figure 7, the performance of the VPA and VKA is almost identical because there is less isolated island space, which causes no significant difference between global and local methods. In general, the number of relay nodes in ATCFS is more than that of the VPA and VKA, while the STFPA needs the least number of relay nodes when the number of islands is less than 20. Since the connection between the VPA and VKA is far away from the island distribution, the four-point connection between the three islands can be approximated as the three-point connection of the triangle when the distribution is sparse, and the STFPA is the shortest connection to the three vertices of the spatial triangle.

When the number of islands exceeds 20, the number of relay nodes required by the STFPA is greater than the VPA and VKA and less than ATCFS. As the number of islands increases, the islands become closer, and the distribution gradually concentrates. The four-point connection of three islands in the VPA and VKA cannot be approximated as a three-point connection of triangles, which makes the STFPA’s optimization not obvious. STFPA gradually approaches ATCFS, but the number of relay nodes is still less than ATCFS.

**Figure 8:**
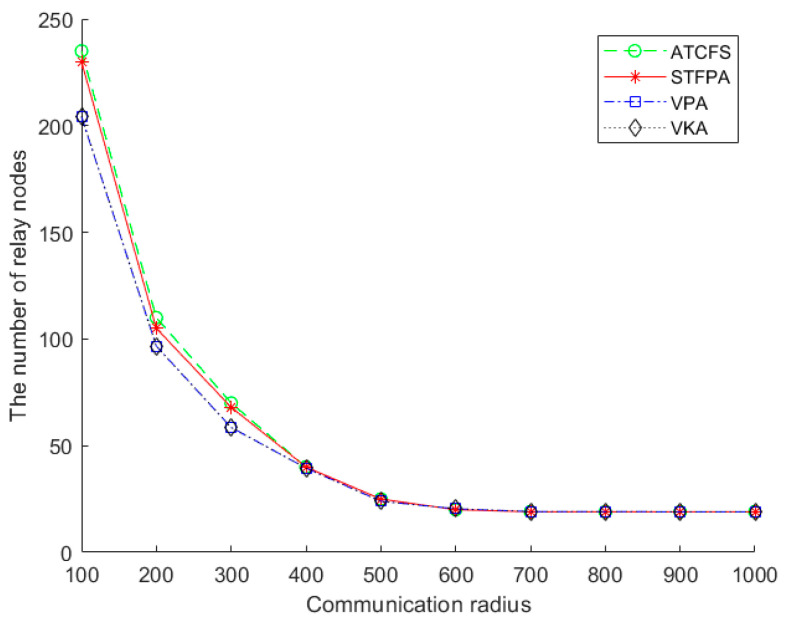
The number of relay nodes vs. the communication radius.

Figure 8 shows the impact of the communication radius on the number of relay nodes. There are 20 boundary sensors on one island, and the number of islands is 20. The number of relay nodes of the STFPA, VPA, VKA, and ATCFS decreases with the increase in communication radius and remains stable after reaching 600. We assume the size of the experimental environment remains unchanged. In that case, the spatial distribution remains unchanged, and the number of relay nodes required to connect the islands will inevitably decrease when the communication radius increases. When the communication radius is greater than 600 and less than 1000, the number of relay nodes remains stable because two to three sensors can connect two to three islands to each other.

#### 5.2.2. The Performance Comparison on the Average Node Degree

**Figure 9:**
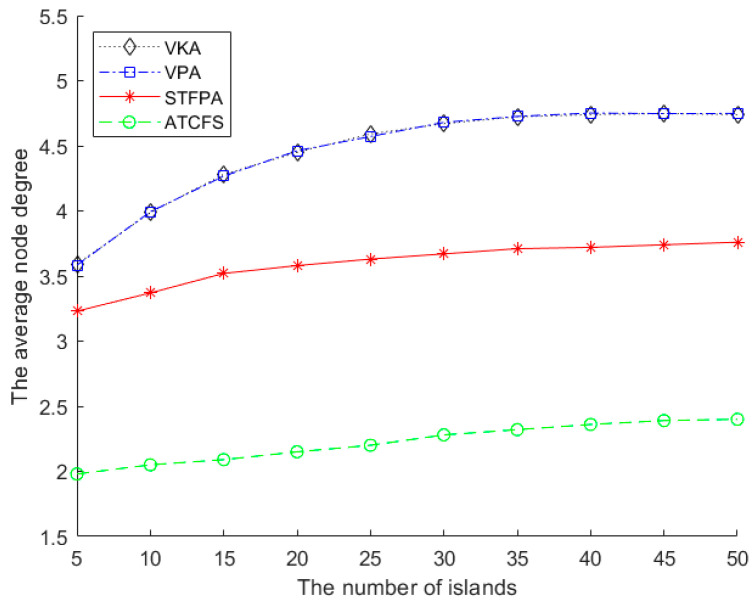
The average node degree vs. the number of islands.

Figure 9 shows the impact of the number of islands on the average node degree. There are 20 boundary sensors on one island, and the communication radius is 500 m. As can be seen from Figure 9, the performance of the VPA and VKA is almost identical because there are fewer isolated islands in this experiment, which causes no significant difference between global and local methods. The average node degree of STFPA is significantly larger than ATCFS and slightly smaller than the VKA and VPA. It increases slowly with the increase in the number of islands. The average node degree of the STFPA, VKA, and VPA proposed in our paper is always higher than ATCFS since we use many boundary nodes to connect each island, meaning the networks restored by our algorithms are more robust than those restored by ATCFS.

**Figure 10:**
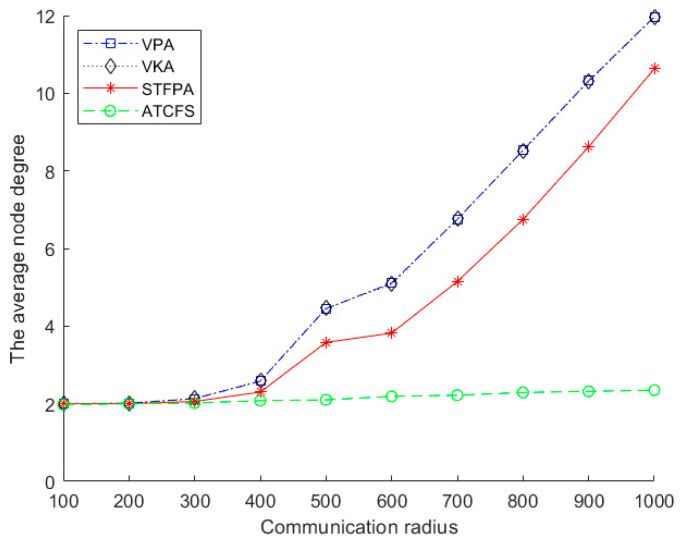
The average node degree vs. the communication radius.

Figure 10 shows the impact of the communication radius on the average node degree. There are 20 boundary sensors on one island, and the number of islands is 20. For the VPA, VKA, STFPA, and ATCFS, the average node degree increases with the increase in communication radius. After the communication radius exceeds 400 m, the VPA, VKA, and STFPA increase sharply, and ATCFS increases slowly. When the communication radius is less than 400 m on the same island, the interconnection among the boundary nodes is sparse, leading to the degree of the boundary nodes being smaller. Meanwhile, the number of relay nodes is larger, and the degree of the relay node is relatively stable. The degree of the relay node of the VPA and VKA is 2, and the degree of the relay node of the STFPA is also 2, except the degree of the relay node placed on the spatial Fermat point is 3. In this circumstance, the degree of average nodes is mainly determined by the degree of relay nodes, leading to a value slightly greater than 2. When the communication radius exceeds 400 m, the mutual connection between boundary nodes gradually increases on the same island. When the communication radius is 1000 m, almost all of them are connected to each other. The node degree of boundary nodes is extremely large, while the number of relay nodes is small. Therefore, the degree of average nodes is mainly determined by the degree of boundary nodes, which leads to a sharp increase.

#### 5.2.3. The Performance Comparison of the Average Hops

**Figure 11:**
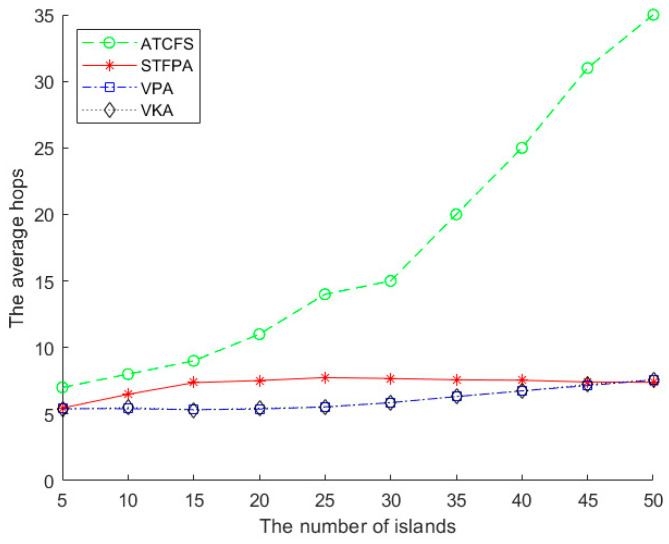
The average hops vs. the number of islands.

Figure 11 shows the impact of the number of islands on the average hops. There are 20 boundary sensors on one island, and the sensing radius is 500 m. As can be seen from Figure 11, ATCFS increases significantly with the increase in the number of islands. The changes in the STFPA, VPA, and VKA are relatively stable. When the number of islands is small, the average hop count of STFPA is slightly higher than that of the VPA and VKA. That is because when the spatial Fermat point replaces the adjacent connection, the replaced points are far away from each other when the number of islands is small. As the number of islands increases, these replaced points will be relatively close.

**Figure 12:**
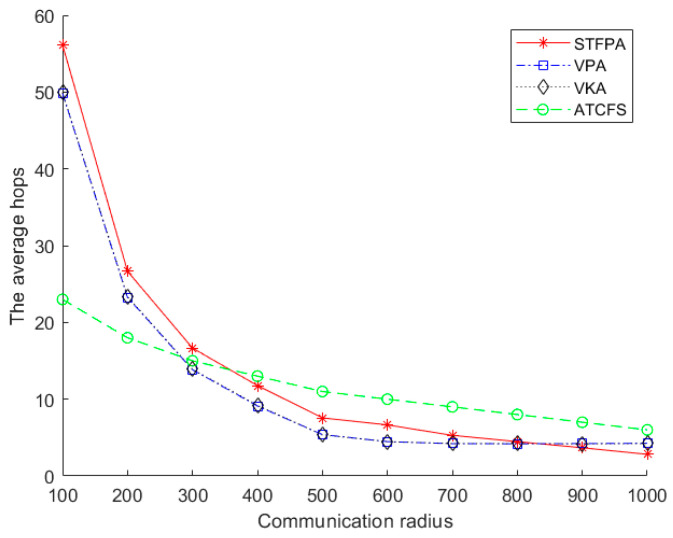
The average hops of different communication radii.

Figure 12 shows the impact of the communication radius on the average hops. There are 20 boundary sensors on one island, and the number of islands is 20. As can be seen from Figure 12, the average hops of the STFPA, VPA, VKA, and ATCFS decrease with the increase in the communication radius. This is because in the same experimental environment and spatial distribution, as the communication radius increases, the number of relay nodes required for the connection between islands will inevitably decrease, resulting in a decrease in the average number of hops. The average number of hops represents the average value of the sum of hops from an island to all other islands, mainly related to the total number of relay nodes and the path’s shape. In the same environment, the number of relay nodes changes with the increase in the communication radius. The change in the number of relay nodes is not obvious under different communication radii. The connection mode of the triangle makes the path shape relatively tortuous when the communication radius is small.

The above simulations and results show that the STFPA, VPA, and VKA methods proposed in this paper are very efficient regarding the number of relay nodes, the average node degree, and the average hops. Our proposed algorithms—the STFPA, VPA, and VKA—perform better than the existing algorithm ATCFS, regardless of the level of network connectivity damage. Compared with ATCFS, our proposed algorithms need fewer relay nodes to restore connectivity. After connectivity restoration, the average node degree is higher, and the average hops are lower in most cases, which means our proposed algorithms construct a more robust network.

The STFPA, VPA, and VKA behave differently in different environments and metrics, which means they suit different environments and meet different application goals. The STFPA has the highest time complexity but requires the least relay nodes when the number of islands is less than 20. The STFPA is more suitable for applications with less island connectivity. Regarding the average node degree and the average hops, the VPA and VKA perform better in most cases, making these two algorithms more suitable for applications with high robustness and high real-time network connectivity recovery.

## 6. Conclusions and Future Work

In this paper, we use the boundary node set to represent the 3D WSNs that need connectivity restoration, define it as a graph connecting problem, and solve it by constructing a minimum spanning tree and a spatial triangle Fermat point Steiner tree. We propose three algorithms, the VKA, VPA, and STFPA, to solve this problem. The results show that the three algorithms are all very efficient regarding the number of relay nodes, the average node degree, and the average hops. The STFPA requires the least relay nodes when the number of islands is less than 20, which makes it more suitable for applications with less island connectivity. The VPA and VKA are more suitable for highly robust applications and real-time network connectivity recovery.

Since our proposed algorithms use the boundary nodes to represent the isolated islands and deploy the relay nodes to connect these boundary nodes to restore connectivity, the boundary node detection algorithm greatly impacts the performance of our proposed algorithms. Now, we use the existing boundary node detection algorithm [33] to determine the boundary node of each island, which may limit the optimizing possibility of relay node deployment. We will develop a coordinated boundary node detection algorithm that considers the intra-island and inter-island information to find the boundary nodes that may be closer to each other to improve the performance further. Based on the performance evaluation, the STFPA, VPA, and VKA perform differently in different environments and suit different optimization goals. Selecting the appropriate algorithm in a real environment may be a challenge. To address this issue, we will develop an adaptive selection strategy to analyze the environment characteristics and select a suitable connectivity restoring algorithm to meet the application constraints and goal. We will also develop the prototype implementations, evaluate them in real environments, and investigate their performance to identify the key parameters further and improve the ultimate performance in future work.

## Figures and Tables

**Table 1:** Summary of existing methods.

Index	3D/2D	Mode	Method	Resource Usage	Disadvantage
[3,4,5,6,7]	2D	Passive		Deploying nodes	Not fully applicable to 3D environments.
[8]	3D/UWSNs	Active		Movement controlled	Not applicable to large-scale damaged networks.
[9]	3D/UWSNs	Active	Clustering/sleep scheduling	Movement controlled	It requires deploying a large number of nodes in advance, leading to resource wastage.
[10]	3D/UWSNs	Active	Clustering	Movement controlled	This method is only suitable for data collection and does not ensure permanent network connectivity.
[11]	3D/UWSNs	Active	Clustering		This method does not detect vulnerabilities or perform connectivity restoration; it only reduces the destruction of sensors by changing the route.
[12]	3D/UWSNs	Active	Clustering and sleep scheduling	Movement controlled	This algorithm does not consider the security of data transmission, primarily how to ensure low energy consumption and high quality of service under various attacks.
[13]	3D/UWSNs	Active	Clustering	Movement controlled	This strategy does not address the error control mechanism during packet transmission.
[14]	3D/UWSNs	Active	Clustering	Movement controlled	Not applicable to large-scale damaged networks.
[15]	3D/UWSNs	Active	Clustering	Movement controlled	It requires deploying many nodes in advance, leading to resource wastage.
[16,17]	3D/UWSNs	Active	Clustering	Movement controlled	The two algorithms are only suitable for the failure of a single node.
[18]	3D/UWSNs	Passive		Deploying nodes	This method’s result could be more optimal. At the same time, when defining the isolated island, one of the locations is also taken as the representative location, but the isolated island may be larger in practice. This will also affect the overall effect of the algorithm.
[19]	3D	Passive		Deploying nodes	One location represents one isolated island, and when deploying relay nodes, the z coordinate of the location is also fixed. The approach’s results could be more optimal.
[20]	3D/UWSNs	Active	Sleep scheduling	Deploying nodes	This method does not consider barriers, coverage holes, and heterogeneous underwater network environments during the connectivity coverage process.
[21]	3D/UWSNs	Active	Sleep scheduling	Movement controlled	This method does not consider the constraints in real scenarios. The sensor node’s sensing range is also affected by environmental changes and multiple obstacle obstructions, and it is variable over time in complex scenarios.
[22]	3D/UWSNs	Active		Movement controlled	This method is unsuitable for a heterogeneous underwater cognitive sensor network, where AUVs lack various sensing and communication capabilities.
[23]	2D	Passive	Clustering	Movement controlled	This method cannot be fully applied in a 3D environment.
[24]	2D	Passive/Active		Movement controlled	This algorithm reduces coverage and cannot be fully applied in a 3D environment.
[25]	2D	Passive	Clustering	Movement controlled	This method needs to consider the issue of restoring connectivity through a limited number of relay nodes and mobile data collectors.
[26]	2D	Passive/Active	Clustering	Movement controlled	It does not consider how to realize k-connectivity restoration in a 3D environment.
[27]	2D	Passive	Clustering and sleep scheduling	Deploying nodes	There is no simulation experiment in the actual scene, and the security of data transmission is not considered. It also cannot be fully applied in a 3D environment.
[28]	2D	Active	Clustering	Movement controlled	This method cannot be fully applied in a 3D environment.

## Data Availability

Dataset available on request from the authors.
